# Malonate Inhibits Virulence Gene Expression in *Vibrio cholerae*

**DOI:** 10.1371/journal.pone.0063336

**Published:** 2013-05-13

**Authors:** Yusuke Minato, Sara R. Fassio, Claudia C. Häse

**Affiliations:** 1 Department of Biomedical Sciences, College of Veterinary Medicine, Oregon State University, Corvallis, Oregon, United States of America; 2 Department of Microbiology, College of Science, Oregon State University, Corvallis, Oregon, United States of America; Laurentian University, Canada

## Abstract

We previously found that inhibition of the TCA cycle, either through mutations or chemical inhibition, increased *toxT* transcription in *Vibrio cholerae*. In this study, we found that the addition of malonate, an inhibitor of succinate dehydrogenase (SDH), decreased *toxT* transcription in *V. cholerae*, an observation inconsistent with the previous pattern observed. Unlike another SDH inhibitor, 2-thenoyltrifluoroacetone (TTFA), which increased *toxT* transcription and slightly inhibited *V. cholerae* growth, malonate inhibited *toxT* transcription in both the wild-type strain and TCA cycle mutants, suggesting malonate-mediated inhibition of virulence gene expression is independent to TCA cycle activity. Addition of malonate also inhibited *ctxB* and *tcpA* expressions but did not affect *aphA*, *aphB*, *tcpP* and *toxR* expressions. Malonate inhibited cholera toxin (CT) production in both *V. cholerae* classical biotype strains O395N1 and CA401, and El Tor biotype strain, N16961. Consistent with previous reports, we confirmed that these strains of *V. cholerae* did not utilize malonate as a primary carbon source. However, we found that the addition of malonate to the growth medium stimulated *V. cholerae* growth. All together, these results suggest that metabolizing malonate as a nutrient source negatively affects virulence gene expression in *V. cholerae*.

## Introduction

*Vibrio cholerae* is the causative agent of cholera, a severe waterborne diarrheal disease. Toxin-coregulated pilus (TCP) and cholera toxin (CT) are essential for its virulence. TCP is a type IV pilus [1] that is required for colonization in the small intestine [2], [3], whereas CT is a potent enterotoxin responsible for inducing cholera symptoms [4]. The expression of TCP and CT are positively regulated by an AraC-type transcriptional factor, ToxT [5], [6]. Transcription of *toxT* is positively regulated by the membrane proteins ToxRS [5] and TcpPH [7]. The expression of *tcpPH* is positively regulated by the AphA [8] and AphB [9] transcriptional regulators.

Malonate is a dicarboxylic acid and a well-known competitive inhibitor of succinate dehydrogenase (SDH). However, it is also known that some bacteria can utilize malonate as a carbon source both aerobically and anaerobically. Malonate decarboxylase is the key enzyme for the degradation of malonate in aerobic malonate-degrading bacteria [10]. Genes encoding malonate decarboxylase (*mdcACDE*) are transcribed in an operon with a gene encoding a malonate transporter (*mdcF*). This operon also includes the *mdcB* gene, which encodes the biosynthesis of a prosthetic group precursor and *mdcG* and *mdcH*, which encode enzymes mediating the transfer of the prosthetic group to the apo-acyl carrier protein (ACP) and transfer of a malonyl residue from malonyl-CoA to the SH moiety of the prosthetic group, respectively.

In a previous attempt to identify negative factors of *toxT* transcription, we found that loss of the primary respiration-linked sodium pump, a NADH:ubiquinone oxidoreductase (NQR), and the TCA cycle related enzymes, Icd, SucA and AspA, resulted in elevated *toxT* expression in *V. cholerae* [7], [11], [12]. These findings suggested a link between central metabolism and virulence gene expression in *V. cholerae*.

In this study, we found that addition of malonate, a known inhibitor of the TCA cycle enzyme SDH, inhibited *toxT* expression and CT production in *V. cholerae*. This observation was unexpected because our previous study had shown the opposite effects of loss of TCA cycle enzymes on *toxT* transcription [12]. Further analysis of this phenomenon revealed that malonate inhibited *toxT* transcription independent to the TCA cycle activities. Although *V. cholerae* did not utilize malonate as a sole carbon source, malonate induced *V. cholerae* growth, prompting us to hypothesize that some malonate metabolizing pathway exists in *V. cholerae* and that some aspect of this pathway negatively affects *toxT* transcription in this organism.

## Materials and Methods

### Bacterial Strains and Growth Conditions

Bacterial strains and plasmids used in this study are listed in Table 1. All bacterial strains were kept at −80°C in 20% glycerol stocks. For β-galactosidase assays, bacterial strains were grown overnight in Luria-Bertani (LB) medium (Difco) at 37°C, washed, diluted to OD_600_ = 0.05 in LB (initial pH 6.5) and then grown for 6 hr at 30°C. Yeast extract peptone water (YEP) was used for the AKI growth condition as described previously [13]. Medium pH was adjusted with HCl. Antibiotics were supplemented as appropriate as follows: streptomycin, 100 µg/ml; and kanamycin, 50 µg/ml. Malonate and NaCl were added to LB (pH 6.5) as indicated. 2-thenoyltrifluoroacetone (TTFA) was added to LB (pH 6.5) at 5 µM.

**Table 1:** Bacterial strains used in this study.

Strains	Description	Source or reference
*V. cholerae*		
O395N1	O1 classical biotype strain, *lacZ* ^−^, Sm^r^	Dr. John Mekalanos
TZ (*toxT*::*lacZ*)	O395N1, *toxT*::*lacZ*, Sm^r^	[7]
TZ*sucA*::TnMar	TZ, *sucA*::TnMar, Sm^r^, Km^r^	[12]
TZ*icd*::TnMar	TZ, *icd*::TnMar, Sm^r^, Km^r^	[12]
TZ*aspA*::TnMar	TZ, *aspA*::TnMar, Sm^r^, Km^r^	[12]
TZnqr	TZ,Δ *nqrA-F*, Sm^r^	[34]
*tcpA*::*phoA*	O395N1, *tcpA*::*phoA*, Sm^r^	[3]
*ctxA*::*phoA*	O395N1, *ctxA*::*phoA*, Sm^r^	[13]
CA401	O1 classical biotype strain, Sm^r^	[35]
N16961	O1 El Tor biotype strain, Sm^r^	Dr. John Mekalanos

### β-galactosidase and Alkaline Phosphatase Assays

β-galactosidase assays were performed as described previously [11]. Alkaline phosphatase assays were performed as described previously [14].

### Quantitative Reverse Transcription-Polymerase Chain Reaction (qRT-PCR) Analysis

qRT-PCR assays were performed essentially as previously described [15]. In brief, cells of *V. cholerae*, grown in LB (initial pH 6.5) at 30°C for 6 hrs, were treated with RNA Protect Bacteria Reagent (Qiagen). RNA was extracted using the QIAGEN RNeasy Mini Kit (Qiagen). Primers used for qRT-PCR are listed in Table 2. Real-time qRT-PCR reactions were performed using the SuperScript® III Platinum® SYBR® Green One-Step qRT-PCR Kit (Invitrogen) and an ABI PRISM 7500 FAST Sequence Detection System (Applied Biosystems) at the OSU CGRB facility.

**Table 2:** DNA primers used in this study.

Primer	Sequence (5′ to 3′)
5Vc16SrRNAqRT	GATCATGGCTCAGATTGAACG
3Vc16SrRNAqRT	TCGCCACCCAAGGAACA
5VcToxTqRT	GCTGTCCTTTCTGAAGTGGTAAATG
3VCToxTqRT	TTCTACTTTCGAGAAGAACCCTGAA
5VcTcpPqRT	GATCCAATGAAGCCGGAAAG
3VcTcpPqRT	CTGATAAATCCATGAGGCCAAAG
5VcToxRqRT	CGGATTAGGACACAACTCAAAAGA
3VCToxRqRT	TGCTTAGGGGATCGAAGGTAAA
5VcAphAqRT	TCAGTACAATCGGCAGAACCTTAC
3VcAphAqRT	TTTCCTGATAGTGAGCGACCAA
5VcAphBqRT	GCACCATCCAATCTGACAAAAC
3VcAphBqRT	GCCAACTCGGAAAATCACATC

### CT-ELISA

CT production was determined by a GM_1_-based enzyme linked immunosorbent assays (CT-ELISA), essentially as described previously [16]. CT-ELISA was performed using a cholera toxin-specific monoclonal antibody (Abcam) and Goat-Anti-Mouse (GAM)-HRP Conjugated antibodies (Bio-Rad). An HRP Substrate kit (Bio-Rad) was used to detect the HRP activity and the plates were read at 415 nm on an iMark microplate reader (Bio-Rad). The amount of CT was quantified using known amounts of purified cholera toxin B subunit (Sigma) as the standard.

## Results

### Malonate Inhibits *toxT*, *ctxB*, and *tcpA* Transcription

**Figure 1:**
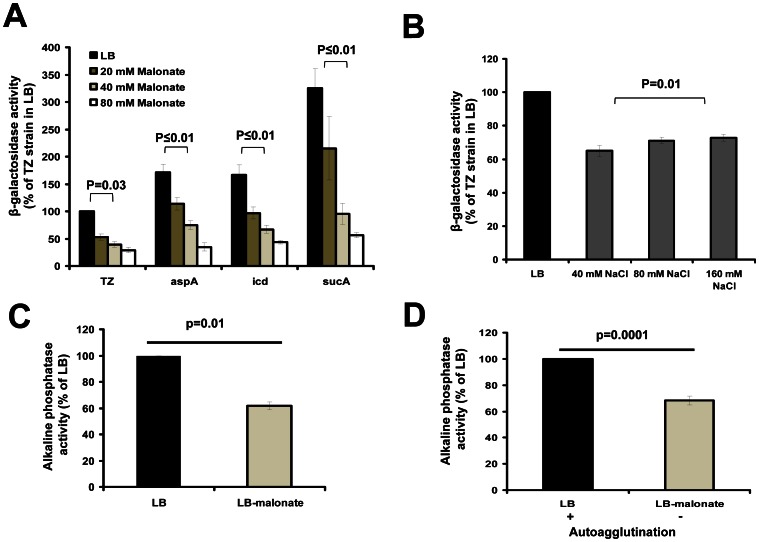
Effects of growth conditions on virulence gene transcriptions. *V. cholerae toxT*::*lacZ* (A and B), *ctx*::*phoA* (C) and *tcpA::phoA* (D) transcription in different growth conditions were measured. Bacteria were inoculated into LB (pH 6.5) and shaken at 30°C for 6 hrs (A and B) or 16 hrs (C and D). Malonate was added to LB (pH 6.5) as indicated (A) or at 40 mM (C and D). NaCl was added to LB (pH 6.5) as indicated (B). Autoagglutination of the *V. cholerae* O395N1 cells was evaluated visually after 16–24 hrs of incubation (“+” means autoagglutination, “−” means no autoagglutination) (D). All experiments were repeated more than three times. The error bars indicate standard deviations. P values were calculated by one-way ANOVA followed by post hoc Tukey test (A and B), or Student’s t test (C and D).

In contrast to our previous finding that inhibition of TCA cycle enzymes increased *toxT* transcription [12], we found that malonate, a potent inhibitor of SDH, significantly inhibited *toxT* transcription of the classical biotype strain of *V. cholerae* O395N1 *toxT*::*lacZ* in a concentration dependent manner (Fig. 1A).

Sodium is also known to affect *V. cholerae toxT* expression [11], [16]. Because we used malonate disodium salt for the *toxT*::*lacZ* measurement, changes in sodium concentration may be responsible for the decreased *toxT* transcription. To test this, we investigated the effect of NaCl on *toxT* transcription. We used 40, 80 and 160 mM NaCl that contained equivalent amounts of sodium ions as the 20, 40 and 80 mM malonate additions. We observed that although NaCl modestly inhibited *toxT* transcription at these concentrations, the effects were significantly lower than those of the malonate (Fig. 1 A and B). In addition, unlike malonate, NaCl did not show concentration-dependent increased inhibition of *toxT* in the concentration range we tested (Fig. 1B). Malonate also inhibited *ctx* and *tcpA* transcription (Fig. 1C and 1D, respectively) further confirming the negative effects of malonate on virulence gene expression in this bacterium.

### Malonate Inhibits *toxT* Independent of the TCA Cycle and ETC Activities

When TCA cycle activity is low, bacteria utilize alternative pathways to generate ATP and to recycle CoASH from acetyl-CoA [17], [18]. One such pathway is the PTA-ACK pathway, which consists of phosphotransacetylase (PTA) and acetate kinase (ACK) [17]–[20]. Metabolic activity through the PTA-ACK pathway results in the excretion of acetate into the external medium [17]. Our previous study revealed that *V. cholerae* TCA cycle mutants (*icd* and *sucA* mutants) and TCA cycle related mutant (*aspA* mutant) showed increased acetate production that also shifted the medium pH to a slightly acidic pH [12]. In addition, we observed that the TCA cycle mutants showed slower growth compared to the parent strain when grown in LB [12]. Similar effects were also observed when SDH was inhibited by another SDH chemical inhibitor, 2-thenoyltrifluoroacetone (TTFA) (data not shown). TTFA increased *toxT* transcription to levels similar to the TCA cycle mutants (data not shown). In contrast, addition of malonate did not affect medium pH and slightly increased the *V. cholerae* growth (data not shown). Such distinguishable differences prompted us hypothesize that malonate affects *toxT* transcription independent to the TCA cycle activity.

To further confirm this idea, we tested the effects of malonate on *toxT* transcription in the TCA cycle mutants. If malonate affects *toxT* expression by affecting TCA cycle activity, the TCA cycle mutants should be insensitive to the addition of malonate. We therefore investigated the effects of malonate on *toxT* transcription in the TCA cycle mutants (*aspA*, *icd* and *sucA*). Consistent with our previous findings, the TCA cycle mutants showed higher *toxT* transcription compared to the parent strain (Fig. 1A). However, malonate still inhibited *toxT* transcription in the TCA cycle mutants (Fig. 1A), indicating that the negative effect of malonate on *toxT* transcription is independent to the TCA cycle activity.

Because SDH is also involved in the electron transport chain (ETC), it could be possible that malonate inhibits *toxT* transcription by inhibiting ETC activity. However, this notion conflicted with our previous finding that inhibition of one of the major ETC components, NQR, increased *toxT* transcription [11]. In addition, similar to the TCA cycle mutants, *toxT* transcription in the Δ*nqrA-F* mutant was sensitive to malonate (data not shown) indicating that the negative effect of malonate on *toxT* transcription is independent to the ETC activity.

### Malonate does not Inhibit Expression of Transcriptional Regulators Operating Upstream of ToxT

**Figure 2:**
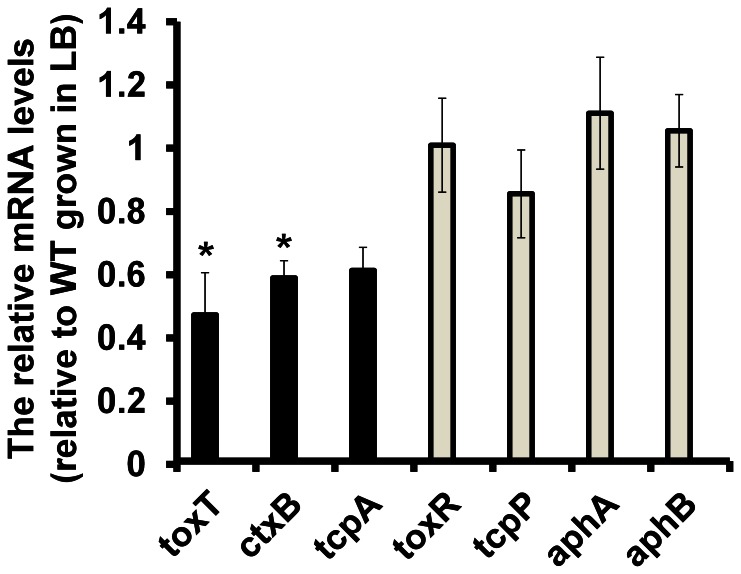
Effect of malonate on virulence gene expressions. *V. cholerae* O395N1 were inoculated into LB (pH 6.5) with or without 40 mM malonate and shaken at 30°C for 6 hrs. Total RNA was extracted and analyzed by qRT-PCR. Gene expression levels were normalized between the samples by using 16S ribosomal RNA. All experiments were repeated three times. The error bars indicate standard deviations. P values were calculated by Student’s t test and *indicates P≤0.05.

To further examine the negative effects of malonate on *V. cholerae* virulence gene expression we performed qRT-PCR analyses. As expected from our *toxT*::*lacZ* analyses, we observed that *toxT* expression levels in *V. cholerae* O395N1 were approximately two-fold lower in the presence of malonate than the control (Fig. 2). Similarly, *ctxB* and *tcpA* were also reduced in the presence of malonate (Fig. 2), as both genes are regulated by ToxT. To investigate if other known transcriptional activators in this regulatory cascade respond to the presence of malonate, we next tested whether malonate also affects *aphA*, *aphB*, *tcpP*, or *toxR* expression. No dramatic effects of malonate were observed on the expression levels of these genes (Fig. 2), however, suggesting that malonate does not appear to affect *toxT* expression by affecting transcription of known regulators functioning upstream of ToxT.

### Malonate Inhibits CT Production in both Classical and El Tor Biotype Strains of *V. cholera*

To confirm the negative effects of malonate on *V. cholerae* virulence, we next investigated the effects of malonate on CT production. Consistent with the gene expression data, addition of malonate inhibits CT production of *V. cholerae* O395N1 strain when tested in LB (pH 6.5) at 30°C (Table 3). We also observed that the *V. cholerae* O395N1 strain did not show an autoagglutination phenotype in LB (pH 6.5) at 30°C (Fig. 1C). Because of the known correlation between the autoagglutination phenotype and TCP production [21], these data indicated that malonate inhibits both of the two major virulence factors in *V. cholerae*.

**Table 3:** CT production of different *V. cholerae* strains under various growth conditions.

Growth conditions	CT production (µg/ml/O.D._600_)		
	O395N1	CA401	N16961
LB	2.2±0.4	3.1±0.1	N.D^a^
LB+40 mM malonate	1.4±0.5	0.8±0.1	N.D^a^
YEP	1.5±0.4	N.D^a^	0.6±0.1
YEP+40 mM malonate	0.5±0.2	N.D^a^	U.D^b^

a not determined.

b undetected.

We then asked whether the effects of malonate on CT production are specific to the *V. cholerae* O395N1 strain. CT production in another *V. cholerae* classical biotype strain, CA401, was also inhibited by malonate, similar to the O395N1 strain (Table 3). To investigate whether malonate also inhibited CT production in an El Tor biotype strain, we tested the effect of malonate on CT production of *V. cholerae* N16961. El Tor biotype strains are required to grow under a specific growth conditions, known as the “AKI conditions” [12], [22], to produce measurable CT production in vitro. *V. cholerae* N16961 produced detectable amounts of CT when grown under AKI growth conditions and addition of malonate to the growth media strongly inhibited CT production (Table 3). We also found that addition of malonate into the medium inhibited CT production of the classical biotype *V. cholerae* strain O395N1 in the AKI condition (Table 3), further demonstrating that the effect of malonate is not growth condition specific. Moreover, the effects of malonate on CT production are not biotype specific.

### *V. cholerae* do not Utilize Malonate as a Sole Carbon Source

Some bacteria are known to utilize malonate as a sole carbon source. Typically, a malonate transporter and malonate decarboxylase are required to utilize malonate. Genetic analysis of the reported *V. cholerae* genomes, including O395 and N16961 strains, revealed that *V. cholerae* do not encode neither a malonate transporter gene (*mdcF*) nor a malonate decarboxylase gene (*mdcA,C,D* and *E*), suggesting that *V. cholerae* do not utilize malonate as the carbon source. Indeed, it was reported that less than 1% of *V. cholerae* strains can utilize malonate as a carbon source [23].

**Figure 3:**
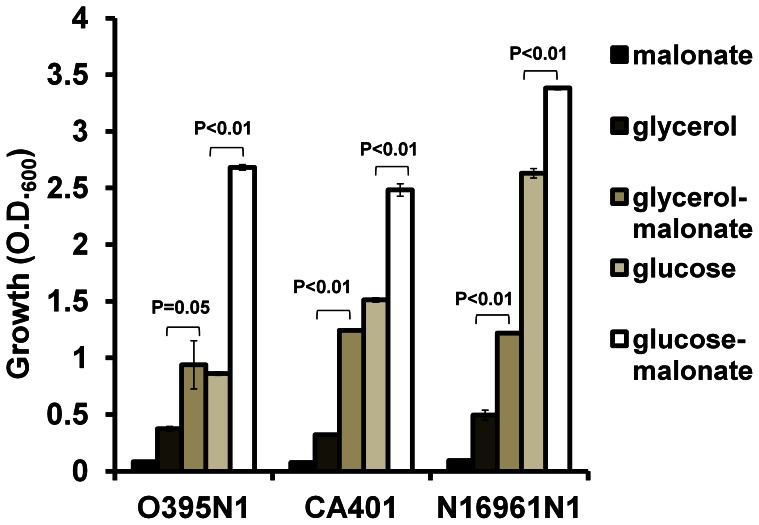
Effects of malonate, glycerol and glucose on *V.cholerae* growth. Bacteria were inoculated into M9 medium supplemented with different carbon sources (f.c. 0.4%). Bacterial growth was measured after 24 hrs shaking. 40 mM malonate was added to the M9-glycerol and M9-glucose media as indicated. All experiments were repeated more than three times. The error bars indicate standard deviations. P values were calculated by Student’s t test.

To confirm this, we performed a growth assay using M9 minimal media supplemented with malonate as the sole carbon source. We observed that all of the *V. cholerae* strains tested did not grow in M9 minimal media supplemented with just malonate (Fig. 3), indicating that these *V. cholerae* strains do not utilize malonate as the sole carbon source.

Although the *V. cholerae* strains we used did not utilize malonate as the sole carbon source, we did notice that the growth of these strains was significantly stimulated when grown in M9 glycerol supplemented with malonate compared to just M9 glycerol (Fig. 3). Similar growth induction by malonate was also observed in M9 glucose (Fig. 3). These observations are consistent with the finding that addition of malonate slightly induced *V. cholerae* growth in LB (see above). Together, these data indicated that *V. cholerae* can utilize malonate as a nutrient source but not as a sole carbon source.

## Discussion

Recent accumulating evidence suggests that metabolism and bacterial virulence are closely related [24]–[26]. In *V. cholerae*, it has been shown that ToxT indirectly inhibits central metabolism pathways, including the TCA cycle and glycolysis [27]. We had previously found that the inhibition of the central metabolic pathway, the TCA cycle and the primary respiration-linked sodium pump, NQR, increased *toxT* transcription in *V. cholerae* [11], [12]. These findings suggest the existence of a feedback loop between central metabolism and ToxT. In addition, other metabolic pathways, such as the Entner-Doudoroff pathway, the anaerobic trimethylamine N-oxide respiration, and methionine metabolism are involved in *V. cholerae* virulence [28]–[30]. Thus, there is a mature link between *V. cholerae* metabolism and virulence. The current study was initiated by the unexpected finding that malonate, a potent SDH inhibitor, inhibited *toxT* expression, whereas inhibition of TCA cycle enzymes by mutations increased *toxT* transcription [12].

When SDH is inhibited, succinate can still be acquired through the glyoxylate shunt. However, we previously showed that the *sucA* and the *icd* mutants showed increased *toxT* transcription [12]. Importantly, these mutants can also acquire succinate through the glyoxylate shunt. Thus, it is unlikely that inhibition of SDH decreases *toxT* transcription because of the glyoxylate shunt. We have also shown that stimulation of the glyoxylate shunt either by adding glyoxylate or acetate does not affect *toxT* transcription [12]. Furthermore, since SDH is also a component of the electron transport chain (ETC), inhibition of SDH inhibits ETC activity. We previously reported that inhibition of a major ETC component of *V. cholerae*, NQR, increased *toxT* transcription [11] but the increased *toxT* was primarly caused by decreased TCA cycle activity [12]. Therefore, it is unlikely that inhibition of SDH decreases *toxT* transcription by affecting ETC activity. Hence, we hypothesized that malonate affects *toxT* independent to TCA cycle activity. To confirm this hypothesis, we tested another SDH inhibitor, 2-thenoyltrifluoroacetone (TTFA). We found that addition of TTFA increased *toxT* transcription, an observation similar to the *toxT* increases observed in the *sucA*, the *icd* and the *aspA* TCA cycle mutants [12]. TTFA also inhibited growth similar to the TCA cycle mutants [12]. In contrast, malonate inhibited *toxT* transcription and slightly induced growth. These data strongly suggested that inhibition of SDH increases *toxT* transcription similar to the TCA cycle mutants and that the effect of malonate on *toxT* is different from the TCA cycle inhibition effect. In addition, we investigated the effects of malonate on several TCA cycle mutants and an ETC mutant (the *nqrA-F* mutant) and found that malonate still inhibited *toxT* transcription in these mutants. These data clearly showed that malonate inhibits *toxT* transcription independent to TCA cycle and ETC activities.

Although, *V. cholerae* did not utilize malonate as the sole carbon source, we observed that addition of malonate, when combined with other carbon sources, induced *V. cholerae* growth, indicating that *V. cholerae* can indeed utilize malonate as a nutrient source. Thus, once inside the *V. cholerae* cells, malonate might be metabolized and changed in structure, thereby no longer being able to inhibit SDH. This concept might explain our conflicting finding that inhibition of TCA cycle decreased *V. cholerae* growth and increased *toxT* transcription because if malonate were to retain its structure inside the *V. cholerae* cells, it would inhibit the TCA cycle and result in decreased growth and increased *toxT* expression.

Three types of enzymes, malonate decarboxylase, malonamidase and malonyl-CoA synthetase, are known to use malonate as their substrate [31]. Malonate decarboxylase is essential for bacterial aerobic utilization of malonate as the sole carbon source. *V. cholerae* strains do not have an ortholog of the known malonate utilizing enzyme complex, malonate decarboxylase (*mdcACDE*) or malonate transporter (*mdcF*). Consistent with this, we observed that *V. cholerae* O395N1 and N16961 strains did not utilize malonate as the sole carbon source. However, our model predicts transport of malonate into *V. cholerae*. The amino acid sequence of *Klebsiella pneumoniae* MdcF transporter is known to show similarity to a *Bacillus subtilis* protein, YwkB [32]. YwkB belongs to the Auxin Efflux Carrier (AEC) Family (TPDB). Interestingly, *V. cholerae* has two genes encoding proteins that belong to the AEC family (VC1229 and VCA0024) and these two proteins also show some similarity to *K. pneumoniae* MdcF. Therefore, it might be possible that *V. cholerae* transports malonate into the cells via these AEC family proteins. We also searched genes that showed similarity with the malonamidase genes in the *V. cholerae* genome but did not find any genes with significant homology. Although we also did not find a true ortholog of malonyl-CoA synthetase from *Rhizobium leguminosarum* (Accession: AAC83455.1) in the *V. cholerae* genome, we found that *V. cholerae* has several genes that showed some amino acid similarity to the malonyl-CoA synthetase (Table 4). It could be possible that malonate is converted to malonyl-CoA by one of these enzymes in *V. cholerae* cells.

**Table 4:** *V. cholerae* predicted acyl-CoA ligases that show similarity to malonyl-CoA synthetase from *Rhizobium leguminosarum.*

VC numbers	Predicted protein functions	Match Values
VC1985	long-chain-fatty-acid–CoA ligase (*fadD*)	4.0e^−53^
VC0772	vibriobactin-specific 2,3-dihydroxybenzoate-AMP ligase (*vibE*)	3.8e^−36^
VC1340	propionate–CoA ligase (*prpE*)	1.1e^−25^
VC0249	RfbL protein (*rfbL*)	6.0e^−21^
VC2484	long-chain-fatty-acid–CoA ligase, putative	1.2e^−17^
VC2341	long-chain-fatty-acid–CoA ligase, putative	2.0e^−17^
VC1971	O-succinylbenzoate–CoA ligase (*menE*)	2.3e^−17^
VC0298	acetyl-CoA synthase (*acs-1*)	2.4e^−17^
VC1579	enterobactin synthetase component F-related protein	3.8e^−16^
VCA1110	long-chain-fatty-acid–CoA ligase, putative	9.0e^−06^
VCA0829	acetyl-CoA synthase (*acs-2*)	2.7e^−3^

**Figure 4:**
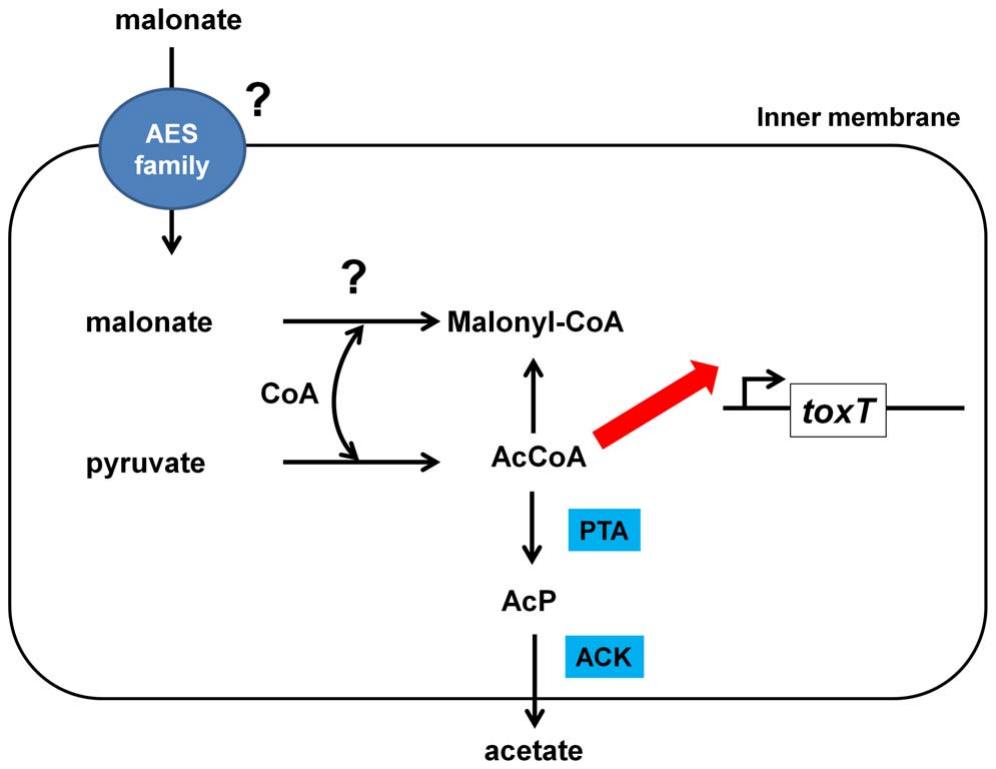
Current working model for the link between malonate and virulence gene expression in *V. cholerae*. Malonate is possibly imported by one of the Auxin Efflux Carrier (AEC) Family proteins (VC1229 and VCA0024). We propose that increased levels of malonyl-CoA decrease the available intracellular CoA levels which results in decreased acetyl-CoA (AcCoA) levels. When TCA cycle activity is low, AcCoA levels should be increased and *V. cholerae* produce acetate from AcCoA and excrete it into the external medium most likely via the PTA and ACK system (see text for more details). Such a link between AcCoA levels and *toxT* transcription, as depicted by the red arrow, has previously been observed [12].

If malonate is indeed converted to malony-CoA, how would such a metabolic change affect *toxT* transcription? As mentioned above, we recently found that inhibition of TCA cycle and NQR, increased *toxT* transcription in *V. cholerae* [11], [12]. These mutants showed increased acetate production and we found that disruption of the major acetate excretion pathway, the PTA-ACK pathway, increased *toxT* transcription. Thus, we concluded that *toxT* transcription is affected by either acetyl-CoA or some metabolic derivative of acetyl-CoA. We are proposing the possibility that malonyl-CoA synthesis from malonate results in the decreased available CoA pool and decreased acetyl-CoA levels (Fig. 4). This idea is consistent with our data that malonate did not affect other virulence related regulators that operate upstream of ToxT. It is also interesting to mention that a *V. cholerae fadD* mutant showed decreased *toxT* expression, indicating that FadD is related to the virulence gene regulation in *V. cholerae* [33]. Although the detailed molecular mechanism is still unclear, lack of FadD impairs the membrane localization of TcpP and results in decreased *toxT* transcription [33]. Similar to the *fadD* mutant, addition of malonate did not affect *tcpP* and *toxR* expression levels. It might be possible that interaction of malonate with FadD modifies its structure and results in impaired TcpP membrane localization.

Together our data demonstrate the intriguing link between malonate and *toxT* expression. Future research is necessary to understand the mechanisms of 1) how malonate is utilized in *V. cholerae* and 2) how such changes in metabolism affect *toxT* expression.
